# Preoperative physical resilience indicators and their associations with postoperative outcomes

**DOI:** 10.1007/s11357-025-01633-6

**Published:** 2025-04-02

**Authors:** Marjolein Klop, René J. F. Melis, G. M. E. E. Geeske Peeters, Guillaume S. C. Geuzebroek, Robin H. Heijmen, Richard J. A. van Wezel, Jurgen A. H. R. Claassen

**Affiliations:** 1 Department of Neurobiology, Donders Institute for Brain, Cognition and Behaviour, Radboud University, Nijmegen, the Netherlands; 2 Department of Geriatric Medicine, Radboud University Medical Center, Nijmegen, the Netherlands; 3 Department of Cardiothoracic Surgery, Radboud University Medical Center, Nijmegen, the Netherlands; 4 OnePlanet Research Center, Radboud University, Nijmegen, the Netherlands; 5 Department of Biomedical Signals and Systems, Technical Medical Centre, University of Twente, Enschede, the Netherlands; 6 Department of Cardiovascular Sciences, University of Leicester, Leicester, UK

**Keywords:** Resilience, Orthostatic blood pressure, Grip strength, Grip work, Postoperative recovery

## Abstract

The health benefit of surgery in older adults may be outweighed by negative effects on cognitive or physical function. Physical resilience is defined as the potential for recovery after a stressor such as surgery. We assessed associations between physical resilience measured by orthostatic blood pressure (BP) and cerebral oxygenation recovery or grip work (sustained hand grip strength; GW) and postoperative outcome in two cohorts of (older) surgical patients. The first cohort (CTC) consisted of patients undergoing complex cardiothoracic surgery. The second cohort (GRR) held geriatric outpatients undergoing various surgical procedures. Outcome measures were length of stay (LoS) and postoperative complications. Negative binomial (LoS) and ordinal (complications) regression models were used to determine associations. 261 patients (113 CTC and 148 GRR) underwent surgery. Median LoS was 10 (CTC) and 5 days (GRR). Postoperative complications occurred in 80% (CTC) and 45% (GRR) of patients. In CTC, 10 mmHg higher systolic BP recovery was associated with a 12% shorter LoS (incidence rate ratio (IRR) 0.88 (95% CI 0.78–0.98)). 10 s longer sustained hand grip was associated with a 5% shorter LoS in GRR (IRR 0.95 (0.90–1.00)), but a 7% longer LoS in CTC (IRR 1.07 (1.03–1.11)). No significant associations were found with postoperative complications. Orthostatic cerebral oxygenation recovery in CTC was not significantly associated with any postoperative outcome. Our results imply that resilience indicators might be associated with LoS after surgery. Future research should seek to replicate our findings and investigate whether adding resilience parameters to preoperative assessment can support postoperative outcome prediction.

**Supplementary Information:**

The online version contains supplementary material available at 10.1007/s11357-025-01633-6.

## Introduction

Although operative risk and mortality increase with age, older patients can still benefit from surgery, including complex cardiothoracic surgery such as a thoracic aortic aneurysm repair [1]. The risks and benefits are weighed using multi-domain information, ideally from a comprehensive geriatric assessment (CGA) [2]. However, uncertainty in clinical outcomes remains, especially for complex surgery [3]. To aid clinical decision-making, information that allows to estimate a patient’s recovery potential may be added to the CGA, which now mainly covers more static parameters like frailty. Furthermore, trained personnel to perform a CGA may not be available at all times and in all settings.

Physical resilience is an individual’s capacity to resist or recover from a stressor, such as surgery [4]. Dynamical coordination and regulation are needed within and between different systems to be resilient to the stressor of surgery. Structural patient characteristics (comorbidities, frailty, body-mass index, etc.) are therefore hypothesized to be insufficient on their own to identify system dynamics and, thus, resilience. Dynamic assessments are needed for resilience quantification, such as an exhaustion, stress, or stimulus–response test [5, 6].

Standing up from a seated or supine position is an example of a stimulus–response test to challenge the cardiovascular system. Impaired orthostatic blood pressure (BP) recovery has already been linked to faster cognitive decline in Alzheimer’s Disease patients and to increased mortality in falls clinic patients [6–8]. More extreme BP impairment when standing up, orthostatic hypotension, is also related to multiple adverse outcomes, such as falls [9], functional decline [10], dementia progression [11], cardiovascular disease [12], and mortality risk [13]. Cerebral oxygenation recovery after a postural change may also be a marker of resilience, as slower recovery has previously been linked to depression [14], slower gait speed [15], and increased multimorbidity [16].

Muscle fatigability is a measure of muscle reserve capacity, which can be measured with a fatigue resistance (FR) test, an example of an exhaustion test, where a participant maximally squeezes a rubber bulb and maintains this for as long as possible [17]. Grip work (GW) can be quantified as the area under the strength-time curve. GW has previously been related to dependency in activities of daily living and self-rated physical functioning in community-dwelling older adults [18, 19].

This study aimed to examine the prospective associations between simple quantitative resilience measures (orthostatic BP recovery and GW indicators) and outcomes after surgery (length of hospital stay and postoperative complications) in patients visiting the cardiothoracic surgery and the geriatric outpatient clinic. We hypothesized that lower resilience would be associated with a longer hospital stay and more postoperative complications. The coordinated analysis of two cohorts (one with a stronger and homogeneous stressor and one with more heterogeneous stressors) was chosen to evaluate the genericness of resilience indicators.

## Methods

### Study population

Two cohorts were included. Cohort 1 (cardiothoracic surgery outpatient clinic, CTC) included patients of all ages planned to undergo elective open thoracic aortic surgery, during their preoperative assessment at the cardiothoracic surgery outpatient clinic of the Radboudumc (Nijmegen, the Netherlands) between February 2020 and September 2022. Participants who were 70 years or older underwent a CGA, including cognitive screening and frailty assessment, while participants younger than 70 years did not, except when the surgeon classified them as frail.

Cohort 2 (geriatric resilience registry, GRR) were patients of 60 years or older referred to the preoperative geriatric outpatient clinic at Radboudumc (Nijmegen, the Netherlands) for various elective surgical procedures, between August 2021 and February 2024.

Exclusion criteria were being unable to understand and follow instructions and being physically unable to perform a supine-stand test or GW test. The studies were performed in accordance with the Declaration of Helsinki. The local ethics committee (CMO Radboudumc) concluded that the studies did not fall within the scope of the Medical Research Involving Human Subjects Act (WMO) and waived formal ethical approval. All participants gave written (CTC) or oral (GRR) informed consent for the use of their medical data for research.

### Data collection

#### Independent variables

All participants performed one postural change: for CTC, a sit-stand transition (5 min sitting – 3 min standing); for GRR, a supine-stand transition (5 min supine – 5 min standing). During this, BP was measured continuously using volume-clamp photoplethysmography (Finapres Medical Systems, Enschede, the Netherlands), worn on the middle finger of the nondominant hand, placed in a sling at heart height. For CTC, cerebral oxygenation was measured simultaneously using near-infrared spectroscopy (NIRS; PortaLite, Artinis Medical Systems, Elst, the Netherlands). Two NIRS sensors were attached to the forehead, approximately 2 cm above the eyebrows, measuring changes in oxygenated (O_2_Hb) and deoxygenated hemoglobin (HHb). The start, moment of standing and end of the measurement were marked using analog pulses by the PortaSync (CTC; Artinis Medical Systems, Elst, The Netherlands) or built-in markers of Finapres (GRR).

All participants performed two (CTC) or three (GRR) maximum grip strength (GSmax) assessments and one GW test, with a rest period of 30 s in between. Participants were stimulated to squeeze the rubber bulb as hard as possible, after which GSmax was noted as the highest value of the repetitions. In the GW tests, patients squeezed the bulb as hard as possible and sustained the strength for as long as possible. The initial strength had to be at least 80% of GSmax measured before. When the grip strength fell below 50% of GSmax, the time in seconds was captured as FR. GW was estimated using the formula GW = GSmax*0.75*FR [19]. The large bulb of a pneumatic dynamometer (Martin Vigorimeter, KLS Martin Group, Tuttlingen, Germany) was used [20].

#### Outcome variables

Outcome variables were length of hospital stay and the Clavien-Dindo complication score, categorizing postoperative complications on a scale of 0 (no complications) to 7 (in-hospital mortality), when deviating from standard care [21]. These outcome parameters were retrieved from electronic patient files. Missing hospital stay due to in-hospital mortality was imputed using the longest length of stay present in the dataset.

#### Covariables

Age, height, weight, multimorbidity (Charlson comorbidity index (CCI) [22]), medication use, surgical procedure, and postoperative complications (3-month mortality, 1-year mortality, reoperation, delirium, bleeding, infection, gastrointestinal complication, renal complication, cardiac arrhythmia, stroke, readmission) were obtained from electronic patient files. Additional data were acquired during the CGA for participants aged ≥ 70 years and frail participants aged < 70 years in the CTC cohort and all participants in the GRR cohort. These included The Older Persons and Informal Caregivers Survey Short Form (TOPICS-SF) measuring daily functioning and quality of life [23], the Montreal Cognitive Assessment (MoCA) as a cognitive screening tool [24], 5-times repeated chair-stand test, clinical frailty scale (CFS) [25], and comprehensive geriatric assessment frailty index (CGA-FI) [26].

### Data acquisition and processing

For CTC, BP and GSmax/GW data were acquired in Acqknowledge (version 5.0, BioPac Systems Inc., Goleta, USA) at 200 Hz. Cerebral oxygenation was recorded in Oxysoft (version 3.0, Artinis Medical Systems, Elst, The Netherlands) at 10 Hz. For GRR, BP data were saved directly on the Finapres device, sampled at 200 Hz. All data were processed in MATLAB (2023b, MathWorks Inc., Natick, USA). BP peak and trough detection was performed using custom-written semi-automatic scripts, resulting in heart rate, diastolic BP (DBP), and systolic BP (SBP) over time, resampled at 10 Hz and filtered with a 5-s moving average filter [27]. Signal quality was assessed visually, and signals of insufficient quality were discarded. For BP, insufficient quality meant the inability to distinguish peaks and troughs. For cerebral oxygenation, insufficient quality was the absence of a visual heartbeat in the O_2_Hb signal for at least 5 s during postural change, a baseline shift larger than 10 µmol/L, or an irregular heartbeat amplitude. All available NIRS channels were filtered using a 5-s moving average and averaged per participant.

From the SBP, DBP and (if present) O_2_Hb curve, recovery at 30–40 s after standing and at 50–60 s after standing were determined by averaging all values between those timepoints. Baseline, defined as the average of 60 to 30 s before standing up, was subtracted from these values.

### Statistical analysis

Statistical analyses were performed in RStudio (2022.02.01, R version 4.1.3). Continuous and ordinal variables are presented as mean (standard deviation) when normally distributed and median [interquartile range] when distributed otherwise. Categorical variables are shown as number (percentage). Two-sided testing was used for all analyses with a significance level of 0.05.

For the primary analysis, associations between resilience parameters (orthostatic BP recovery and GW indicators) and outcome parameters were investigated using negative binomial regression for length of hospital stay and ordinal logistic regression for the Clavien-Dindo complication score. In the secondary analyses, we repeated all regression analyses using cerebral oxygenation recovery as an independent variable. All model results were obtained using complete samples and after multiple imputation for resilience parameters. BP recovery values, GW indicators, and oxygenation recovery parameters were missing completely at random when caused by logistic reasons, and BP recovery values were missing at random when the BP measurement failed (see Supplementary Table S1). The latter can be due to high vessel stiffness, peripheral vascular disease, and cold or arthritic fingers, which are more common in frail, older participants with more comorbidities, for which CFS, age, and CCI were taken as predictors for multiple imputation [28]. Multiple imputation was performed using Multiple Imputation by Chained Equations (MICE), creating ten datasets, and results were pooled using Rubin’s Rule [29].

All models were reported adjusted for age and gender (model 1), and additionally for dichotomized CFS (0 for CFS < 4 (‘very fit’ to ‘managing well’) and 1 for CFS ≥ 4 (‘vulnerable’ to ‘severely frail’)) and CCI without age (model 2). Frailty was not documented for those CTC patients < 70 years old who did not have a consultation with a geriatrician. They were assumed to be non-frail (dichotomized CFS of 0). Model 1 and 2 were additionally adjusted for the complexity of surgery in the GRR cohort. The complexity of surgery was expressed as the odds of mortality of a specific surgical category, which was classified based on the Dutch classification of surgical procedures [30]. For instance, the odds ratio for mortality was 2.49 after aortic surgery and 0.08 after knee surgery (referenced to the mean score of all types of surgery). When a patient underwent multiple surgical procedures in one session, the highest odds ratio was taken. If a surgery did not fall within one of the categories, a low score (mean of all odds ratios in the sample < 1), middle score (odds ratio of 1), or high score (mean of all odds ratios in the sample > 1) was taken as an estimate in consultation with a physician (JC). The logarithm of this score was taken as a covariable in the models. Continuous resilience parameters and covariables considered in the models were centered on the sample mean to facilitate the interpretation of model estimates. BP recovery parameters, GSmax, and FR were scaled by division by a factor of 10 and GW by a factor of 100. All models were tested and reported for CTC and GRR separately in a coordinated analysis [31].

## Results

### Baseline characteristics

CTC consisted of 120 patients, of whom 113 (94%) actually underwent surgery. They had a median age of 65 years. GRR included 253 geriatric outpatients, of whom 148 (59%) underwent surgery with a median age of 78 years (Table 1). In CTC, most patients underwent open thoracic aortic surgery, while surgical procedures in GRR were more varied. The median [interquartile range (IQR)] odds of postoperative mortality of the surgical categories included in the cohorts were 2.49 [2.49–2.49] for CTC and 2.49 [0.56–2.49] for GRR, relative to the average of all surgical procedure categories [30].

**Table 1:** Study cohorts and characteristics of participants and surgeries

	CTC (*n *= 120)		GRR (*n* = 253)	
Study protocol				
Setting	Cardiothoracic surgery outpatient clinic		Preoperative geriatric outpatient clinic	
Postural change type	Sit-stand		Supine-stand	
NIRS measurement	Yes		No	
Participant characteristics	*n*		*n*	
Refrained from surgery^a^, n (%)	120	7 (6)	249	101 (41)
Underwent surgery^b^, n (%)	120	113 (94)	249	148 (59)
Males, n (%)	113	79 (70)	148	156 (62)
Age (years), median [IQR]	113	65 [56–72]	148	78 [74–82]
BMI (kg/m^2^), mean (SD)	113	26.4 (3.9)	147	27.4 (4.4)
MoCA, mean (SD)	45	25.2 (2.5)	136	24.6 (3.5)
5 × chair-stand (s), median [IQR]	44	12 [10–14]	141	14 [11–18]
Gait speed (m/s), median [IQR]	46	1.0 [0.8–1.2]	121	1.0 [0.9–1.2]
CFS, median [IQR]	46	3 [2–4]	148	4 [3–5]
CGA-FI, median [IQR]	44	0.10 [0.06–0.16]	148	0.14 [0.90–0.20]
Baseline SBP (mmHg), mean (SD)	111	134 (22)	148	148 (22)
Baseline DBP (mmHg), mean (SD)	111	83 (12)	148	81 (12)
Comorbidities				
CCI, median [IQR]	113	1 [1–2]	148	2 [1–4]
Hypertension, n (%)	113	60 (53)	148	85 (57)
Cerebrovascular disease, n (%)	113	13 (12)	148	28 (19)
Diabetes, n (%)	113	3 (3)	148	28 (19)
Heart failure, n (%)	113	62 (55)	148	23 (16)
Connective tissue disease, n (%)	113	16 (14)	148	8 (5)
COPD, n (%)	113	14 (12)	148	22 (15)
Myocardial infarction, n (%)	113	14 (12)	148	22 (15)
Kidney disease, n (%)	113	5 (4)	148	18 (12)
Medication				
Number of drugs, mean (SD)	113	6.0 (3.3)	148	8.4 (4.4)
Antihypertensives, n (%)	113	94 (83)	148	106 (72)
Beta blockers, n (%)	113	64 (68)	148	63 (60)
Statins, n (%)	113	56 (50)	148	87 (59)
Antidepressants, n (%)	113	9 (8)	148	15 (10)
Antipsychotics, n (%)	113	2 (2)	148	3 (2)
Benzodiazepines, n (%)	113	9 (8)	148	20 (14)
Surgery	*n*		*n*	
Time to surgery (days)^c^, median [IQR]	113	69 [38–108]	148	74 [24–133]
Surgical complexity^d^, median [IQR]	113	2.49 [2.49–2.49]	148	2.49 [0.56–2.49]
Vascular – aortic^e^, n (%)	113	108 (96)	148	56 (38)
Open aortic arch, n (%)		15 (14)		1 (2)
Open ascending aortic, n (%)		53 (49)		-
Open descending thoracic aortic, n (%)		40 (37)		3 (6)
Vascular—peripheral, n (%)	113	-	148	2 (1)
Cardiac—valvular, n (%)	113	30 (27)	148	2 (1)
Cardiac—CABG, n (%)	113	6 (5)	148	-
Cardiac—other, n (%)	113	7 (6)	148	-
Abdominal—intestinal, n (%)	113	-	148	6 (4)
Abdominal—gastric, n (%)	113	-	148	1 (1)
Abdominal—esophagus, n (%)	113	-	148	4 (3)
Abdominal—pancreatic, n (%)	113	-	148	4 (3)
Abdominal—spleen, n (%)	113	-	148	1 (1)
Abdominal—liver, n (%)	113	-	148	4 (3)
Orthopedic—hip, n (%)	113	-	148	20 (14)
Orthopedic—knee, n (%)	113	-	148	3 (2)
Urologic—bladder, n (%)	113	-	148	8 (5)
Urologic—renal, n (%)	113	-	148	5 (3)
Urologic—renal transplant	113	-	148	1 (1)
Breast, n (%)	113	-	148	1 (1)
Brain, n (%)	113	-	148	3 (2)
Gynecology, n (%)	113	-	148	12 (8)
Adrenal, n (%)	113	-	148	2 (1)
Lung nontransplant, n (%)	113	-	148	1 (1)
ENT, n (%)	113	-	148	7 (5)
Other, n (%)	113	1 (1)	148	9 (6)

^a^Missing for four patients in the GRR cohort, as a decision about surgery has not been made yet. They were excluded from further analyses

^b^Patients who underwent more than one type of surgery, for example a combined aortic aneurysm and heart valve repair, were counted for each indication and thus multiple times in this table

^c^Number of days between preoperative assessment and surgery

^d^Complexity of surgery expressed as the odds of mortality, relative to all surgical procedures [30]

^e^In the CTC cohort almost all patients underwent open thoracic aortic surgery, which is why these procedures were subdivided into aortic arch, ascending aortic and descending aortic repair

Categorical variables are presented as number (percentage) and continuous variables as mean (standard deviation) or median [interquartile range], as appropriate. *n* represents the total number of patients with known data. NIRS: near-infrared spectroscopy, CTC: cardiothoracic surgery outpatient clinic, GRR: geriatric resilience registry, BMI: body mass index, MoCA, Montreal Cognitive Assessment, scaled from 0 to 30, with higher values indicating better cognitive performance, CFS: clinical frailty scale, scaled from 0 to 10 with higher values indicating more frailty, CGA-FI: comprehensive geriatric assessment-frailty index, scaled from 0 to 1, where higher values indicate more frailty, CCI: Charlson comorbidity index, scaled from 0 to 33, with higher values indicating more comorbidities, SBP: systolic blood pressure, DBP: diastolic blood pressure, COPD: chronic obstructive pulmonary disease, ENT: ear-nose-throat, CABG: coronary artery bypass grafting

### Data availability

Of continuous BP data, 76 (GRR) to 91% (CTC) were available for patients who underwent surgery (Supplementary Figure S1). GW indicators were available in more than 95% of the cases, and continuous cerebral oxygenation data in 78% of CTC participants.

### Resilience parameters

**Fig. 1:**
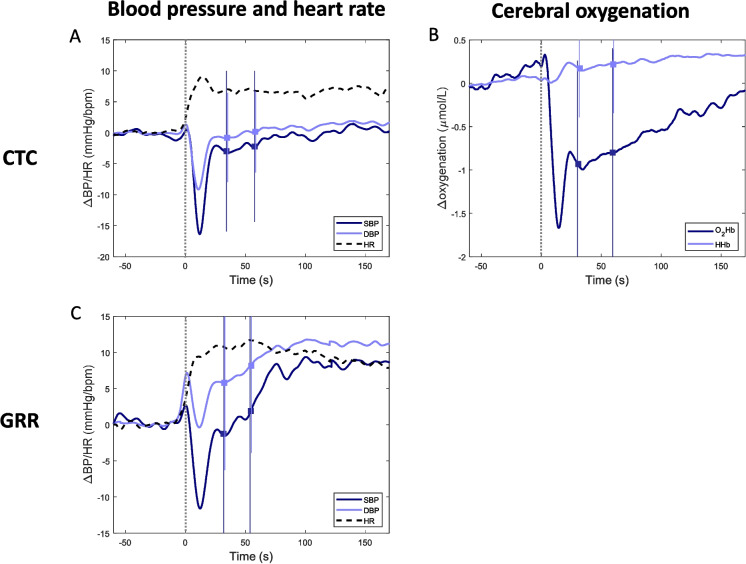
Average courses of blood pressure (BP) and heart rate (HR; **A + C**) and cerebral oxygenation (**B**) of patients who underwent surgery in different cohorts. **A + B**: cardiothoracic surgery outpatient clinic (CTC), **C**: geriatric outpatient clinic (GRR). **A + C**: systolic BP (SBP) in dark blue, diastolic BP (DBP) in light blue, HR in dashed black. **B**: oxygenated hemoglobin (O_2_Hb) in dark blue, deoxygenated hemoglobin (HHb) in light blue. Courses are shown from 1 min before to 170 s after standing up from a supine (**C**) or sitting (**A + B**) position. A grey dotted vertical line indicates the moment of standing up. The mean and standard deviation are shown around 35 s and 55 s after standing up, with a square and line respectively

Figure 1 shows the average BP, HR, and oxygenation course after postural change per group of participants. Both cohorts showed a clear BP drop after standing, followed by an average recovery to (CTC) or above (GRR) baseline levels. The average or median GSmax and GW were higher in the CTC than GRR cohort and higher for males than females in both cohorts (Table 2). FR was more comparable across cohorts and across males and females.

**Table 2:** Grip strength, grip work, and fatigue resistance for patients undergoing surgery from the cardiothoracic surgery outpatient clinic (CTC) and geriatric resilience registry (GRR), stratified for sex

	CTC				GRR			
	Male		Female		Male		Female	
	n	mean (SD)/ median [IQR]	n	mean (SD)/ median [IQR]	n	mean (SD)/ median [IQR]	n	mean (SD)/ median [IQR]
Grip strength (kPa)	77	84.1 (22.0)	33	54.1 (13.1)	96	73.2 (21.9)	52	48.4 (11.7)
Grip work (kPa s)	74	2927 [1600–4320]	33	1625 [838–2807]	96	2228 [1437–3571]	52	1304 [999–2414]
Fatigue resistance (s)	74	47.7 [27.3–68.8]	33	47.4 [28.9–78.8]	96	47.8 [31.7–70.5]	52	47.0 [28.4–64.3]

### Outcome parameters

One-year mortality was 4% for CTC and 9% for GRR (Table 3). The median hospital stay was shorter for GRR (5 days) than CTC (10 days). All but one CTC participant was admitted to the ICU, compared to 32% in the GRR cohort. Complications occurred in 80% of CTC patients, most often delirium. In the GRR cohort, 55% of the patients had an uncomplicated postoperative course.

**Table 3:** Postoperative outcome parameters

Outcomes after surgery	CTC (n = 113)		GRR (n = 148)	
	*n*		*n*	
Mortality				
Within 3 months, number (%)	113	4 (4)	148	3 (2)
Within 1 year, number (%)	113	5 (4)	148	13 (9)
Hospital stay (days), median [IQR]	110	10.0 [7.3–16.0]	139	5.0 [2.0–9.0]
Estimated hospital stay (days), median [IQR]^a^	113	10.0 [8.0–17.0]	142	5.0 [2.0–9.3]
ICU admission, number (%)	113	112 (99)	143	45 (32)
ICU stay (days), median [IQR]^b^	110	1.0 [1.0–3.0]	42	2.0 [1.0–4.0]
Estimated ICU stay (days), median [IQR]^c^	112	1.0 [1.0–3.0]	45	2.0 [1.0–4.0]
Complications	*n*	number (%)	*n*	number (%)
Reoperation^d^	113	11 (10)	143	2 (1)
Delirium	113	30 (27)	143	10 (7)
Infection^e^	113	28 (24)	143	17 (11)
Bleeding^f^	113	8 (7)	143	7 (5)
CVA/TIA	113	7 (6)	143	0 (0)
Permanent damage	113	3 (2)	143	0 (0)
Renal complications^g^	113	12 (11)	143	6 (4)
Hemodialysis	113	5 (4)	143	0 (0)
Gastrointestinal complications^h^	113	3 (2)	143	8 (5)
Cardiac arrhythmia	113	30 (27)	143	7 (5)
Readmission	110	12 (11)	140	9 (6)
Clavien-Dindo classification				
No complication	113	22 (19)	143	78 (55)
Grade I – no pharmacological or surgical interventions	113	20 (18)	143	16 (11)
Grade II – pharmacological interventions	113	38 (34)	143	24 (17)
Grade IIIa – intervention under regional anesthesia	113	7 (6)	143	5 (3)
Grade IIIb – intervention under general anesthesia	113	5 (4)	143	3 (2)
Grade IVa – intensive care for single-organ dysfunction	113	9 (8)	143	11 (8)
Grade IVb – intensive care for multi-organ dysfunction	113	9 (8)	143	3 (2)
Grade V – in-hospital mortality	113	3 (3)	143	3 (2)

CTC: cardiothoracic surgery outpatient clinic, GRR: geriatric resilience registry, ICU: intensive care unit, CVA: cerebrovascular accident, TIA: transient ischemic attack

*n* represents the total number of patients with known data

^a^Patients who died during their stay were given the longest stay present in the cohort. Length of hospital stay for one GRR patient was missing due to a transfer to another hospital

^b^All patients who were admitted to the ICU and did not die during their stay at the ICU

^c^Patients who died during their ICU stay were given the longest stay present in the cohort

^d^Reoperation was defined as a rethoracotomy in the CTC cohort and as a reoperation similar to the original operation in the GRR cohort (e.g., pleural drainage or pericardiocentesis not included)

^e^All types of infections, e.g., pneumonia, urinary tract infection, wound infection, sepsis

^f^Bleeding was defined as hemoglobin decrease needing blood transfusion or reoperation

^g^All types of renal complications, e.g., acute or chronic renal failure, renal infarction

^h^All types of gastrointestinal complications, e.g., gastroparesis, paralytic ileus, pancreatitis, diverticulitis

In CTC, lower BP recovery, especially at 50–60 s, was associated with a longer hospital stay (incidence rate ratio (IRR) of 0.88 (95% CI 0.78–0.98), such that for each 10 mmHg decrease in BP recovery, hospital stay was 12% longer), adjusted for age and sex. These associations attenuated after additional adjustments for CCI and frailty (model 2), covariables associated with hospital stay (model 1). No significant associations were found in the GRR cohort (Table 4). There were no statistically significant associations between BP recovery and complications in both cohorts (Table 4). Similar associations were found in complete-case analyses with narrower confidence intervals (Supplementary Table S2 and S3).

**Table 4:** Preoperative resilience parameters and their association with length of hospital stay after surgery and postoperative complications expressed by the Clavien-Dindo complication score (0 no complications – 7 mortality)

Length of stay	CTC (*n* = 113)				GRR (*n* = 143)			
	Model 1		Model 2		Model 1		Model 2	
Resilience parameter	IRR (CI)	*p*	IRR (CI)	*p*	IRR (CI)	*p*	IRR (CI)	*p*
SBP recovery 30–40 s (*10 mmHg)	0.90 (0.81–1.00)	0.063	0.96 (0.87–1.07)	0.446	0.95 (0.87–1.03)	0.215	0.95 (0.88–1.03)	0.214
SBP recovery 50–60 s (*10 mmHg)	0.88 (0.78–0.98)	**0.031**	0.93 (0.83–1.04)	0.199	0.96 (0.89–1.04)	0.299	0.97 (0.89–1.04)	0.355
DBP recovery 30–40 s (*10 mmHg)	0.85 (0.69–1.04)	0.116	0.94 (0.78–1.14)	0.550	0.90 (0.77–1.06)	0.215	0.90 (0.77–1.05)	0.194
DBP recovery 50–60 s (*10 mmHg)	0.80 (0.64–0.99)	**0.045**	0.89 (0.73–1.08)	0.247	0.90 (0.80–1.04)	0.171	0.90 (0.78–1.05)	0.177
Grip strength (*10 kPa)	1.00 (0.93–1.08)	0.994	1.01 (0.93–1.08)	0.808	1.05 (0.96–1.16)	0.289	1.05 (0.95–1.15)	0.345
Grip work (*100 kPa s)	1.01 (1.00–1.02)	**0.016**	1.01 (1.00–1.02)	**0.006**	0.99 (0.98–1.00)	0.097	0.99 (0.98–1.00)	0.084
Fatigue resistance (*10 s)	1.07 (1.03–1.11)	**0.001**	1.07 (1.03–1.11)	**0.001**	0.95 (0.90–1.00)	**0.049**	0.95 (0.90–1.00)	**0.043**
Covariables								
CCI	1.18 (1.06–1.33)	**0.005**			1.07 (0.99–1.15)	0.073		
Frailty	2.06 (1.40–3.04)	** < 0.001**			0.73 (0.46–1.18)	0.200		
Complication score								
Resilience parameter	OR (CI)	*p*	OR (CI)	*p*	OR (CI)	*p*	OR (CI)	*p*
SBP recovery 30–40 s (*10 mmHg)	1.01 (0.78–1.30)	0.950	1.04 (0.80–1.34)	0.779	0.99 (0.85–1.17)	0.946	1.00 (0.85–1.18)	0.991
SBP recovery 50–60 s (*10 mmHg)	0.94 (0.71–1.26)	0.694	0.97 (0.72–1.30)	0.845	0.99 (0.85–1.16)	0.936	1.00 (0.85–1.18)	0.982
DBP recovery 30–40 s (*10 mmHg)	1.05 (0.64–1.72)	0.838	1.09 (0.66–1.78)	0.747	0.92 (0.69–1.22)	0.565	0.93 (0.70–1.24)	0.621
DBP recovery 50–60 s (*10 mmHg)	0.98 (0.57–1.68)	0.937	1.02 (0.59–1.76)	0.936	0.95 (0.72–1.26)	0.714	0.96 (0.73–1.28)	0.792
Grip strength (*10 kPa)	0.94 (0.77–1.13)	0.490	0.94 (0.77–1.14)	0.515	1.08 (0.89–1.31)	0.444	1.08 (0.89–1.32)	0.424
Grip work (*100 kPa s)	1.01 (0.99–1.03)	0.392	1.01 (0.99–1.03)	0.302	0.99 (0.97–1.01)	0.169	0.99 (0.97–1.01)	0.177
Fatigue resistance (*10 s)	1.10 (0.98–1.24)	0.095	1.11 (0.99–1.24)	0.077	0.93 (0.84–1.04)	0.193	0.93 (0.83–1.04)	0.198
Covariables								
CCI	1.10 (0.82–1.49)	0.526			1.05 (0.91–1.21)	0.515		
Frailty	2.31 (0.74–7.18)	0.153			1.10 (0.45–2.73)	0.833		

Negative binomial regression models (length of hospital stay) and ordinal logistic regression models (complication score) for the cohort at the cardiothoracic surgery outpatient clinic (CTC) and geriatric outpatient clinic (GRR). Model 1: adjusted for age and sex. Model 2: adjusted for age, sex, frailty, and Charlson comorbidity index (CCI). Models for GRR were additionally adjusted for surgical complexity. All continuous and ordinal variables were centered. Grip strength, fatigue resistance, and orthostatic blood pressure variables were scaled by a factor 10 and grip work by a factor 100. IRR: incidence rate ratio, OR: odds ratio, CI: 95% confidence interval. Missing resilience parameters were imputed using multiple imputation. P-values < 0.05 are indicated in bold

Higher FR and GW were significantly associated with shorter hospital stay in GRR (IRR 0.95 (0.90–1.00) for each 10 s increase in FR and 0.99 (0.98–1.00) for a 100 kPa s increase in GW), but with longer hospital stay in the CTC cohort (IRR 1.07 (1.03–1.11) for each 10 s increase in FR and 1.01 (1.00–1.02) for a 100 kPa s increase in GW; Table 4). Higher FR showed a trend (p < 0.1) towards more complications in the CTC cohort, but no other significant associations or trends were found between GW indicators and complication score (Table 4). In complete-case analyses, similar associations were found (Supplementary Table S2 and S3).

Oxygenation recovery values were not significantly associated with any of the outcome values (Supplementary Table S4).

## Discussion

Stimulus–response and exhaustion physical resilience measures were determined in 373 patients from two preoperative cohorts (CTC and GRR), with 261 patients undergoing surgery. Strongest associations with postoperative outcomes were observed in the CTC cohort, which faced the most impactful and homogeneous stressor, cardiothoracic surgery. Here, slower BP recovery was associated with a longer hospital stay. Adding the covariables multimorbidity and frailty to our model attenuated this association, suggesting that BP recovery is related to multimorbidity and frailty. In GRR, the point estimates for BP recovery were in the same direction, but generally smaller and not statistically significant. Lower GW and FR were associated with longer hospital stay in GRR. Contrary to expectations, in CTC, higher GW and FR were associated with a longer hospital stay. No significant associations were found between physical resilience indicators and postoperative complications.

Previous research into preoperative resilience measurements often focused on psychological resilience using scales like the brief resilience scale, with mixed associations with outcome after surgery [32–34]. However, these studies mostly assessed patient-reported outcome measures such as quality of life, in contrast to the more physical postoperative outcomes in our study. Resilience is outcome-specific, meaning that a similar stressor (in this case surgery) can exhibit a different level of resilience in the psychological and physical domain. Findings from psychological resilience studies are therefore not directly comparable to our study, although physical and psychological resilience are likely influencing each other [35]. For instance, the number of stressful life events has been related to impaired orthostatic SBP recovery [36].

Impaired BP recovery, indicative of sub-optimal BP regulation, has been associated with adverse outcomes that might reflect lower resilience, such as faster cognitive deterioration [8], mortality [7], accelerated brain aging [37], and frailty [15]. However, this relationship has not extensively been evaluated before a known stressor, such as elective surgery. Preoperative orthostatic hypotension has been associated with a longer postoperative hospital stay [38] and postoperative complications like nausea and vomiting [39]. Lower BP complexity, related to poor BP regulation [40], has been associated with a higher risk of adverse cardiovascular outcomes after surgery [41]. Our findings of an association between lower BP recovery and longer hospital stay in the CTC cohort may thus fit in a pattern suggesting that BP dysregulation may be negatively associated with recovery from physical stressors. In the GRR cohort, we only found a trend (p < 0.1) for similar associations in the complete-case analysis. An explanation may be that in CTC all patients underwent cardiovascular surgery; in the GRR cohort, less than 50% of the included surgical procedures were cardiovascular. The orthostatic challenge directly tests the cardiovascular system and may better reflect recovery after cardiovascular surgery. In the GRR cohort, approximately 25% of the orthostatic BP measurements were missing. Similar findings in the imputed and complete-case analyses suggest little influence of the missing orthostatic BP data on the associations with outcomes after surgery. However, the large proportion of missing BP data indicates that orthostatic BP with this continuous BP device is less feasible for individual resilience quantification in a frailer cohort, compared to the less-frail CTC cohort where only 9% of BP measurements were missing mostly for logistical reasons. The cohorts also differed at group level in their BP response, with the GRR cohort showing an overshoot which could indicate that more patients in that cohort had orthostatic hypertension. How orthostatic hypertension affects the use of BP recovery as a resilience measure could be a relevant topic for future research. Furthermore, integrating multiple resilience measures should be investigated in association with outcomes after various types of surgery, such as in the GRR cohort. Cerebral oxygenation recovery is highly related to BP recovery [42], and was therefore expected to be associated with outcome parameters. However, BP recovery was already weakly associated, and oxygenation measurements were available for fewer participants and are a more indirect measure of the orthostatic BP response, because the effect of BP on oxygenation can be reduced by intact cerebral autoregulation, possibly explaining the lack of associations.

GW has been shown to be lower for pre-frail than robust older adults [43]. Preoperative GW has not been investigated in relation to surgical outcomes before. GW has been shown to decrease and then recover after surgery and be associated with surgery-induced inflammation [44]. The more static parameter GSmax has been extensively investigated before surgery. Lower GSmax has often, but not consistently, been associated with adverse outcomes like a longer hospital stay and postoperative morbidity [45], for instance after abdominal surgery [46] or gastrectomy [47]. In our study, GSmax did not show any associations with surgical outcome after correction for age and sex, known correlates of GSmax [48]. Our results suggest that FR and GW are associated with length of hospital stay, although opposite associations were observed between the cohorts. The finding that longer FR was related to longer hospital stay was in contrast with our hypothesis and seems counterintuitive. This finding in CTC could be due to unforeseen bias. In FR measurements, also mental factors influence how long someone can sustain grip. In CTC, participant may have released grip before exhaustion due to factors such as perceived rush due to the tight schedule of outpatient clinic appointments, or the fear to cause aneurysm rupture, as most aneurysm patients had received strict advise to avoid strenuous activities. However, we are unable to verify whether these measurement-related issues could fully explain the opposite association between FR and length of stay, between GRR and CTC. Future studies in different cohorts could further elucidate this association.

Various factors might have contributed to somewhat inconsistent and weaker associations between the physical resilience indicators and postoperative outcomes. First, patients were carefully selected for surgery based on characteristics such as frailty, comorbidities, age, and the complexity of the planned surgery. In the CTC cohort, only relatively fit older adults were referred (median CFS of 3). The GRR cohort included frailer older adults (median CFS of 4) than the CTC cohort. This can be explained by selection, because, depending on the type of surgery, the surgeon specifically referred these patients to a geriatrician for an additional preoperative assessment of their fitness for surgery. In addition to being more frail, the GRR cohort also had a higher median age (difference of 13 years). Together, the higher age and frailty reflect the distinct patient populations. The GRR cohort is more heterogeneous, as the decision to undergo minor surgery is less impactful than the decision to undergo, for example, open aortic surgery, while both cases are represented in this cohort. Different resilience measures may be more suited depending on frailty. Our results suggest that BP recovery can be an indicator in the hypothesized direction in a less frail cohort (CTC). In contrast, GW indicators behaved as hypothesized in a frailer cohort (GRR). Second, the time between preoperative assessment and surgery was not constant and ranged from one day to more than a year, depending on waiting lists, the COVID-19 pandemic, personal circumstances, or surgery requiring improved fitness. As a result of preoperative advice (no smoking, healthy eating, exercising), resilience might have improved after preoperative assessment and before surgery. Especially orthostatic BP recovery is known to be variable [49]. Future research, including repeated pre-surgery measures, ideally also shortly before surgery, is recommended to verify current findings. Third, the Clavien-Dindo complication score considers the most severe complication that occurred but does not sum multiple (mild) complications, making its responsivity to mild complications limited. On the other hand, length of hospital stay indirectly takes multiple complications into account. This might explain why associations were found with length of stay but not with complication score.

A strength of this study is the application of quantitative physical resilience measures in two real-world cohorts without strict exclusion criteria, enhancing generalizability and including external validation by exploring associations in both data sets separately. There are some limitations to this study. First, participants underwent different surgical procedures, impacting the severity of the stressor. We corrected for surgical complexity in the GRR cohort using odds of mortality risk from a large Dutch registry [30]. However, surgical categories were still clustered. This meant, for example, that an abdominal aortic aneurysm repair and an aortic arch aneurysm repair (often endovascular surgery in GRR) were both considered ‘aortic surgery’, while the latter, mainly present in the CTC cohort, is higher risk surgery. This makes both cohorts less comparable than they seem to be based on the surgical complexity score that we used and supports our coordinated analysis approach. Second, for practical reasons, the CTC cohort included a sit-stand orthostatic BP test instead of a supine-stand, which might overestimate BP recovery [50]. Third, we only used short-term outcomes in this study (in-hospital follow-up), which depend on the complexity and course of the surgical procedure, which was homogeneous and invasive in CTC, but less so in GRR. This is illustrated by more complications and a longer median hospital stay in CTC. Ideally, these short-term outcome measures should be complemented with functional recovery measures at multiple timepoints to capture the recovery trajectory, including information on whether functional recovery was reached at home or not. Length of stay is an indirect measure of recovery, i.e. functioning well enough to leave the hospital. However, it is influenced by the discharge destination or availability of home care and is thus not purely indicative of recovery. Additionally, each type of surgery requires a minimal length of hospital stay determined by protocols. We corrected for this by using the surgical complexity score (the odds for mortality), and indeed more complex surgeries had a longer length of stay (OR (95% CI) 1.34 (1.16–1.56)), but this adjustment may not fully capture the same aspects.

Physical resilience assessment by orthostatic BP recovery and GW indicators is not yet applicable in clinical practice to indicate a patient’s recovery potential and thus risk of surgery, as some results were inconclusive and we did not assess predictive value. Future studies should try to replicate our findings in larger cohorts, including additional outcomes such as daily functioning and postoperative mortality. If the predictive value of resilience measures beyond currently used predictors can be confirmed, resilience measures could complement the CGA. Additionally, our results suggest overlap between BP recovery and frailty and multimorbidity, implying potential to explore patient selection for CGA based on resilience measures rather than age.

In conclusion, physical resilience parameters were associated with length of hospital stay, suggesting their potential to capture resilience to a surgical stressor. However, associations for GW indicators were inconsistent across both cohorts, and adjustment for known predictors of postoperative outcome, frailty and multimorbidity, attenuated the associations for BP recovery parameters. Further research should investigate the predictive value of these and other resilience parameters to determine whether they can complement or partially replace the CGA and support clinical decision-making.

## Supplementary Information

Below is the link to the electronic supplementary material.

Supplementary file1 (DOCX 54 KB)

## Data Availability

Data will be made available upon reasonable request to the corresponding author.
