# Supplementary material for: Preoperative physical resilience indicators and their associations with postoperative outcomes
Source: GeroScience. 2025 Apr 2;47(3):4857–70. doi: 10.1007/s11357-025-01633-6 (PMC12181507; doi:10.1007/s11357-025-01633-6)
Supplement: Supplementary file 1 — Supplementary file1 (DOCX 54 KB) [file 11357_2025_1633_MOESM1_ESM.docx]

**Supplementary Material -** **Preoperative physical resilience indicators and their associations with postoperative outcomes**

Marjolein Klop, René J.F. Melis, G.M.E.E. (Geeske) Peeters, Guillaume S.C. Geuzebroek, Robin H. Heijmen, Richard J.A. van Wezel, Jurgen A.H.R. Claassen

Contents:

A: Data availability for resilience parameters

B: Baseline characteristics of participants with missing and non-missing continuous blood pressure measurements.

C: Complete-case analyses

D: Associations for cerebral oxygenation

**A: Data availability for resilience parameters**

**Figure S1.** Flowchart of the included resilience parameters, numbers are presented for all participants who had surgery. Logistic reasons for the absence of data are for example the unavailability of a measurement device, time constraints to perform the full assessment, or data loss. GRR: geriatric resilience registry, CTC: cardiothoracic surgery outpatient clinic, BP: blood pressure, NIRS: near-infrared spectroscopy, TSI-FF: tissue saturation index-fit factor.

**B: Characteristics of participants with missing and non-missing continuous blood pressure measurements.**

**Table S1.** Baseline characteristics and outcome variables for patients with missing compared to non-missing blood pressure measurements. Categorical variables presented as number (%), continuous/ordinal variables as median [interquartile range] or mean (standard deviation).

|  | **CTC** |  |  | **GRR** |  |  |
| --- | --- | --- | --- | --- | --- | --- |
|  | **Non-missing (n=103)** | **Missing (n=10)** | **p-value** | **Non-missing (n=113)** | **Missing (n=35)** | **p-value** |
| Males | 71 (70) | 8 (80) | 0.713 | 74 (66) | 22 (63) | 0.935 |
| Age (years) | 65 [57-72] | 62 [55-70] | 0.424 | 77 (6) | 79 (7) | 0.068 |
| BMI (kg/m^2^) | 26.5 (3.9) | 25.8 (4.0) | 0.615 | 27.4 (4.4) | 27.4 (4.2) | 0.996 |
| MoCA | 25.2 (2.5) | 26.3 (2.3) | 0.437 | 24.5 (3.7) | 24.9 (2.8) | 0.569 |
| 5x chair-stand (s) | 12 [10-14] | 14 [13-14] | 0.124 | 13.5 [10.9-17.3] | 13.3 [12.4-18.8] | 0.277 |
| Gait speed (m/s) | 1.0 [0.9-1.2] | 0.9 [0.9-1.0] | 0.236 | 1.1 (0.3) | 0.9 (0.3) | 0.013 |
| CFS | 3.0 [2.0-3.3] | 4.0 [3.5-4.0] | 0.124 | 3.6 (1.2) | 4.4 (1.1) | 0.001 |
| CGA-FI | 0.10 [0.06-0.15] | 0.12 [0.08-0.16] | 0.907 | 0.13 [0.09-0.18] | 0.15 [0.12-0.23] | 0.083 |
| Baseline SBP (mmHg) | 134 (22) | 138 (15) | 0.539 | 149 (23) | 142 (19) | 0.094 |
| Baseline DBP (mmHg) | 82 (12) | 87 (12) | 0.300 | 83 (12) | 77 (10) | 0.010 |
| Comorbidities |  |  |  |  |  |  |
| CCI | 1.0 [1.0-2.0] | 1.0 [1.0-2.0] | 0.953 | 2 [1-3] | 3 [1-5] | 0.030 |
| Hypertension | 56 (54) | 4 (40) | 0.591 | 64 (57) | 21 (60) | 0.876 |
| Cerebrovascular disease | 13 (13) | 0 (0) | 0.500 | 19 (17) | 9 (26) | 0.354 |
| Diabetes | 2 (2) | 1 (10) | 0.629 | 22 (20) | 6 (17) | 0.952 |
| Heart failure | 56 (54) | 6 (60) | 0.993 | 18 (16) | 5 (14) | 1.000 |
| Connective tissue disease | 15 (15) | 1 (10) | 1.000 | 6 (5) | 2 (6) | 1.000 |
| COPD | 12 (12) | 2 (20) | 0.793 | 17 (15) | 5 (14) | 1.000 |
| Myocardial infarction | 12 (12) | 2 (20) | 0.793 | 16 (14) | 6 (17) | 0.872 |
| Kidney disease | 5 (5) | 0 (0) |  | 12 (11) | 6 (17) | 0.462 |
| Medication |  |  |  |  |  |  |
| Number of drugs | 6.0 (3.1) | 6.3 (4.6) | 0.748 | 8.5 (4.6) | 8.1 (3.7) | 0.611 |
| Antihypertensives | 86 (84) | 8 (80) | 1.000 | 81 (72) | 25 (71) | 1.000 |
| Beta blockers | 59 (70) | 5 (63) | 1.000 | 47 (59) | 16 (64) | 0.815 |
| Statins | 51 (50) | 5 (50) | 1.000 | 67 (59) | 20 (57) | 0.977 |
| Antidepressants | 7 (7) | 2 (20) | 0.389 | 9 (8) | 6 (17) | 0.211 |
| Antipsychotics | 2 (2) | 0 (0) | 1.000 | 3 (3) | 0 (0) | 0.774 |
| Benzodiazepines | 8 (8) | 1 (10) | 1.000 | 15 (13) | 5 (14) | 1.000 |
| **Other independent variables** | |  |  |  |  |  |
| GSmax | 75 (25) | 75 (17) | 0.989 | 65.1 (21.8) | 62.3 (24.0) | 0.507 |
| GW | 2211 [1267-3949] | 3023 [2883-4014] | 0.203 | 1894 [1263-3232] | 1800 [1061-3163] | 0.464 |
| FR | 45.7 [25.4-67.6] | 78.8 [56.9-90.7] | 0.091 | 46.9 [29.9-68.0] | 47.7 [27.7-69.5] | 0.884 |
| **Outcome variables** | |  |  |  |  |  |
| Length of stay, days | 10.0 [7.8-16.0] | 10.5 [7.5-24.3] | 0.598 | 5.0 [2.0-9.0] | 6.0 [2.2-8.8] | 0.429 |
| Complication score |  |  | 0.172 |  |  | 0.797 |
| 0, n (%) | 21 (20) | 1 (10) |  | 58 (54) | 20 (57) |  |
| I, n (%) | 19 (18) | 1 (10) |  | 13 (12) | 3 (9) |  |
| II, n (%) | 34 (33) | 4 (40) |  | 20 (19) | 4 (11) |  |
| IIIa, n (%) | 7 (7) | 0 (0) |  | 3 (3) | 2 (6) |  |
| IIIb, n (%) | 4 (4) | 1 (10) |  | 2 (2) | 1 (3) |  |
| IVa, n (%) | 9 (9) | 0 (0) |  | 7 (7) | 4 (11) |  |
| IVb, n (%) | 6 (6) | 3 (30) |  | 3 (3) | 0 (0) |  |
| V, n (%) | 3 (3) | 0 (0) |  | 2 (2) | 1 (3) |  |

CTC: cardiothoracic surgery outpatient clinic, GRR: geriatric resilience registry, BMI: body mass index, MoCA, Montreal Cognitive Assessment, scaled from 0 to 30, with higher values indicating better cognitive performance, CFS: clinical frailty scale, scaled from 0 to 10 with higher values indicating more frailty, CGA-FI: comprehensive geriatric assessment-frailty index, scaled from 0 to 1, where higher values indicate more frailty, CCI: Charlson comorbidity index, scaled from 0 to 33, with higher values indicating more comorbidities, SBP: systolic blood pressure, DBP: diastolic blood pressure, COPD: chronic obstructive pulmonary disease, ENT: ear-nose-throat, CABG: coronary artery bypass grafting, GSmax: maximum grip strength, GW: grip work, FR: fatigue resistance.

**C: Complete-case analyses**

**Table S2** Preoperative resilience parameters and their association with length of hospital stay.

| **Length of stay** | **CTC**  **n=103/107** |  |  |  | **GRR**  **n=113/148** |  |  |  |
| --- | --- | --- | --- | --- | --- | --- | --- | --- |
|  | **Model 1** |  | **Model 2** |  | **Model 1** |  | **Model 2** |  |
| **Resilience parameter** | IRR (CI) | p | IRR (CI) | p | IRR (CI) | p | IRR (CI) | p |
| SBP recovery 30-40 s (* 10 mmHg) | 0.89 (0.80-0.98) | **0.015** | 0.95 (0.86-1.05) | 0.266 | 0.95 (0.87-1.03) | 0.240 | 0.95 (0.87-1.03) | 0.254 |
| SBP recovery 50-60 s (* 10 mmHg) | 0.86 (0.77-0.96) | **0.005** | 0.91 (0.82-1.02) | 0.076 | 0.96 (0.89-1.03) | 0.282 | 0.96 (0.89-1.04) | 0.353 |
| DBP recovery 30-40 s (* 10 mmHg) | 0.83 (0.66-1.04) | **0.044** | 0.95 (0.77-1.16) | 0.546 | 0.88 (0.7-1.001) | 0.087 | 0.87 (0.76-1.00) | 0.074 |
| DBP recovery 50-60 s (* 10 mmHg) | 0.77 (0.61-0.98) | **0.008** | 0.88 (0.70-1.10) | 0.183 | 0.88 (0.77-1.01) | 0.098 | 0.88 (0.77-1.01) | 0.100 |
| Grip strength (*10 kPa) | 1.00 (0.93-1.07) | 0.960 | 1.01 (0.94-1.08) | 0.834 | 1.06 (0.96-1.17) | 0.238 | 1.05 (0.95-1.16) | 0.285 |
| Grip work (*100 kPa s) | 1.01 (1.00-1.02) | **0.017** | 1.01 (1.00-1.02) | **0.005** | 0.99 (0.98-1.00) | 0.093 | 0.99 (0.98-1.00) | 0.081 |
| Fatigue resistance (*10 s) | 1.07 (1.03-1.12) | **0.001** | 1.07 (1.03-1.12) | **0.001** | 0.95 (0.90-1.00) | **0.043** | 0.95 (0.90-1.00) | **0.035** |
| **Covariables** |  |  |  |  |  |  |  |  |
| CCI | 1.18 (1.06-1.32) | **0.004** |  |  | 1.07 (0.99-1.16) | 0.071 |  |  |
| Frailty | 2.06 (1.41-3.09) | **<0.001** |  |  | 0.73 (0.46-1.21) | 0.197 |  |  |

Numbers are presented as n=available for blood pressure analysis/available for grip work analysis. Negative binomial regression models for the cohort at the cardiothoracic surgery outpatient clinic (CTC) and geriatric outpatient clinic (GRR). Model 1: adjusted for age and sex. Model 2: adjusted for age, sex, frailty, and Charlson comorbidity index (CCI). Models for GRR were additionally adjusted for surgical complexity. All continuous and ordinal variables were centered. Grip strength, fatigue resistance, and orthostatic blood pressure variables were scaled by a factor 10 and grip work by a factor 100. IRR: incidence rate ratio, CI: 95% confidence interval. P-values <0.05 are indicated in bold.

**Table S3**. Preoperative resilience parameters and their association with postoperative complications expressed by the Clavien-Dindo complication score (0 no complications – 7 mortality).

| **Complication score** | **CTC**  **n=103/107** |  |  |  | **GRR**  **n=113/148** |  |  |  |
| --- | --- | --- | --- | --- | --- | --- | --- | --- |
|  | **Model 1** |  | **Model 2** |  | **Model 1** |  | **Model 2** |  |
| **Resilience parameter** | OR (CI) | p | OR (CI) | p | OR (CI) | p | OR (CI) | p |
| SBP recovery 30-40 s (*10 mmHg) | 1.00 (0.78-1.28) | 0.997 | 1.03 (0.80-1.32) | 0.834 | 0.98 (0.81-1.17) | 0.787 | 0.99 (0.82-1.19) | 0.891 |
| SBP recovery 50-60 s (*10 mmHg) | 0.92 (0.69-1.23) | 0.582 | 0.95 (0.71-1.26) | 0.708 | 0.95 (0.80-1.12) | 0.543 | 0.96 (0.81-1.14) | 0.672 |
| DBP recovery 30-40 s (*10 mmHg) | 1.04 (0.62-1.69) | 0.870 | 1.08 (0.64-1.75) | 0.775 | 0.89 (0.63-1.24) | 0.477 | 0.91 (0.64-1.27) | 0.566 |
| DBP recovery 50-60 s (*10 mmHg) | 0.97 (0.54-1.64) | 0.900 | 1.01 (0.56-1.72) | 0.980 | 0.89 (0.64-1.23) | 0.479 | 0.91 (0.65-1.27) | 0.569 |
| Grip strength (*10 kPa) | 0.93 (0.77-1.13) | 0.480 | 0.94 (0.77-1.14) | 0.504 | 1.10 (0.90-1.33) | 0.359 | 1.10 (0.90-1.34) | 0.341 |
| Grip work (*100 kPa s) | 1.01 (0.99-1.03) | 0.445 | 1.01 (0.99-1.03) | 0.336 | 0.99 (0.97-1.01) | 0.178 | 0.99 (0.97-1.01) | 0.172 |
| Fatigue resistance (*10 s) | 1.10 (0.98-1.23) | 0.108 | 1.11 (0.99-1.24) | 0.085 | 0.94 (0.84-1.05) | 0.259 | 0.94 (0.83-1.04) | 0.242 |
| **Covariables** |  |  |  |  |  |  |  |  |
| CCI | 1.10 (0.81-1.50) | 0.526 |  |  | 1.05 (0.91-1.22) | 0.509 |  |  |
| Frailty | 2.30 (0.76-7.45) | 0.153 |  |  | 1.02 (0.39-2.51) | 0.963 |  |  |

Numbers are presented as n=available for blood pressure analysis/available for grip work analysis. Ordinal logistic regression models for the cohort at the cardiothoracic surgery outpatient clinic (CTC) and geriatric outpatient clinic (GRR). Model 1: adjusted for age and sex. Model 2: adjusted for age, sex, frailty, and Charlson comorbidity index (CCI) added as covariables. Models for GRR were additionally adjusted for surgical complexity. All continuous and ordinal variables were centered. Grip strength, fatigue resistance, and orthostatic blood pressure variables were scaled by a factor 10 and grip work by a factor 100. OR: odds ratio, CI: 95% confidence interval. P-values <0.05 are indicated in bold.

**D: Associations for cerebral oxygenation**

**Table S4**. Cerebral oxygenation (O_2_Hb) parameters in association with outcome parameters (length of stay, as determined using negative binomial regression, and Clavien-Dindo complication score, using ordinal logistic regression).

|  | **Model 1** |  | **Model 2** |  |
| --- | --- | --- | --- | --- |
| **Multiple imputation (n=113)** | OR (CI)/IRR (CI) | p | OR (CI)/IRR (CI) | p |
| **Length of stay** |  |  |  |  |
| O_2_Hb 30-40 s (µmol/L) | 0.99 (0.85-1.15) | 0.899 | 1.02 (0.88-1.18) | 0.833 |
| O_2_Hb 50-60 s (µmol/L) | 1.02 (0.88-1.17) | 0.841 | 1.04 (0.90-1.21) | 0.571 |
| **Complications** |  |  |  |  |
| O_2_Hb 30-40 s (µmol/L) | 1.05 (0.76-1.44) | 0.771 | 1.05 (0.76-1.45) | 0.735 |
| O_2_Hb 50-60 s (µmol/L) | 1.13 (0.82-1.58) | 0.457 | 1.12 (0.82-1.62) | 0.421 |
| **Complete-case analysis (n=88)** | OR (CI)/IRR (CI) | p | OR (CI)/IRR (CI) | p |
| **Length of stay** |  |  |  |  |
| O_2_Hb 30-40 s (µmol/L) | 1.01 (0.90-1.14) | 0.810 | 1.03 (0.92-1.15) | 0.618 |
| O_2_Hb 50-60 s (µmol/L) | 1.06 (0.94-1.19) | 0.328 | 1.07 (0.96-1.19) | 0.264 |
| **Complications** |  |  |  |  |
| O_2_Hb 30-40 s (µmol/L) | 1.11 (0.81-1.51) | 0.530 | 1.07 (0.77-1.47) | 0.677 |
| O_2_Hb 50-60 s (µmol/L) | 1.23 (0.90-1.71) | 0.198 | 1.21 (0.88-1.68) | 0.251 |

Model 1: adjusted for age and sex. Model 2: adjusted for age, sex, frailty, and Charlson comorbidity index (CCI) added as covariables. Oxygenation variables were centered. IRR: incidence rate ratio, OR: odds ratio, CI: 95% confidence interval. P-values <0.05 are indicated in bold.
